# RBP-J and ITAM crosstalk

**DOI:** 10.18632/oncotarget.6034

**Published:** 2015-10-08

**Authors:** Baohong Zhao

**Affiliations:** Arthritis and Tissue Degeneration Program and David Z. Rosensweig Genomics Research Center, Hospital for Special Surgery and Department of Medicine, Weill Cornell Medical College, New York, NY, USA

**Keywords:** RBP-J, osteoclast, ITAM, bone, arthritis

Osteoclasts, multinucleated giant cells derived from the monocyte/macrophage lineage, are the exclusive specialized bone-resorbing cells that are indispensable for skeletal development, bone remodeling and repair and mineral homeostasis. Increased osteoclastogenesis leads to excessive bone resorption in osteoporosis and in inflammatory diseases with bone destruction, such as rheumatoid arthritis (RA), psoriatic arthritis (PsA), juvenile chronic arthritis (JCA) and periodontitis [1]. As bone is one of the major metastatic sites for tumorigenesis of the breast, prostate, lung as well as multiple myeloma, abnormal generation and activation of osteoclasts facilitate skeletal metastasis and tumor growth in bone tissue [2]. Thus, appropriate control of osteoclastogenesis will be a direct and highly effective approach in the prevention and treatment of osteolysis in pathological conditions.

The immunoreceptor tyrosine-based activation motif (ITAM)-mediated signaling pathways play important roles in various cellular activities, including immune response and cancer activation. The main ITAM-containing adaptors expressed by myeloid osteoclast precursors are DNAX-activating protein 12 (DAP12) and Fc receptor common γ subunit (FcRγ). These adaptors associate with and mediate signaling by various receptors, including DAP12-associated triggering receptor expressed in myeloid cells 2 (TREM2) and signal-regulatory protein β 1 (SIRPβ1), FcRγ-associated osteoclast-associated receptor (OSCAR), paired immunoglobulin-like receptor-A (PIR-A) and FcRs. Osteoclasts require ITAM-mediated costimulation of RANK signaling for their appropriate differentiation during bone homeostasis [3]. Osteoclastogenesis is delicately modulated at various levels, but little is known about the mechanisms that regulate ITAM-mediated costimulation. Recombinant recognition sequence binding protein at the J_κ_, site (RBP-J, also named RBP-J_κ_,, CSL or CBF1) functions as a central transcription factor that receives inputs from several signaling pathways, such as the canonical Notch pathway, Wnt-β-catenin, NF-κB pathway, TLR and TNF signaling pathways. We recently identified RBP-J as a key negative regulator that imposes the requirement for ITAM-mediated costimulation of osteoclastogenesis, homeostatic bone remodeling and inflammatory bone resorption, and discovered the signaling pathways and mechanisms by which RBP-J regulates ITAM-mediated costimulation [4, 5].

Loss of DAP12 or both DAP12 and FcRγ in mice leads to severe osteopetrosis due to impaired osteoclastogenesis and resorptive function. To investigate the role of RBP-J in ITAM-mediated bone remodeling, we generated DAP12 and RBP-J double knockout (KO) mice (R-DKO) and DAP12, FcRγ and RBP-J triple KO mice (TKO) [5]. Strikingly, we found that RBP-J deficiency almost completely reversed the defects of osteoclast differentiation program and significantly rescued the osteopetrotic bone phenotype of *Dap12* KO or *Dap12/Fcrg* double knockout (DKO) mice by bypassing the requirement for costimulation of osteoclastogenesis during bone homeostasis. In inflammatory settings, RBP-J deficiency enabled TNF-α to induce osteoclast formation and bone resorption in *Dap12* KO mice. These data show that RBP-J deficiency allows osteoclast differentiation to occur independently of ITAM-mediated costimulation during homeostatic bone remodeling and inflammatory bone destruction. Thus, RBP-J imposes the requirement for ITAM-mediated costimulation. During osteoclastogenesis, ITAM-mediated PLCγ2-calcium signaling is constitutively active at basal level, which provides essential costimulatory signals for RANK signaling to induce osteoclast differentiation. We show that RBP-J restrains ITAM signaling and limits crosstalk between RANK and ITAM associated receptors by suppressing PLCγ2 expression and keeping PLCγ2-calcium signaling in a repressed basal state. This is the first study showing the negative regulation of ITAM signaling by RBP-J. The regulatory mechanisms of PLCγ2 expression were unclear. Our results show that the mechanism of PLCγ2 regulation is transcriptional in osteoclast differentiation. Furthermore, built upon RNAseq experiments, we identified a novel regulatory loop whereby RBP-J restrains the expression of TGFβR1 and thereby modulates cell responsiveness to TGF-β, which in turn regulates transcription of PLCγ2 [5].

Our findings provide evidence for a cross talk between RBP-J and ITAM-mediated signaling pathways in osteoclast precursors and suggest a conceptually new model where ITAMs positively while RBP-J negatively regulates PLCγ2-calcium signaling. The balance between these opposing pathways fine tunes osteoclastogenesis and determines the strength of activating signals required for osteoclastogenesis in physiological and various pathological settings. For example, we observed that RBPJ expression level was significantly suppressed in synovial fluid macrophages derived from RA patients, indicating that RBP-J expression can be altered in response to environmental cues in pathological states [5]. As RBPJ is a newly identified risk locus for RA, our data providing new evidence for a link between RBP-J and RA highlights the significance of understanding RBP-J mediated mechanisms in osteoclastogenesis and bone resorption. The cross talk between RBP-J and ITAM-mediated signaling pathway as well as the signaling network identified by our study may also exist in other cell types. In cancers, Notch/RBP-J is directly involved in abnormal differentiation of myeloid cells, including dendritic cells and myeloid-derived suppressor cells (MDSC) that can function as osteoclast progenitors to enhance bone loss in tumors [6, 7]. Therefore, the newly described RBP-J/PLCγ2/calcium pathway would also have therapeutic implications for abnormal osteoclastogenesis and associated skeletal destruction in the cancer setting.
